# Correction: Does the Integration of Migrants in the Host Society Raise COVID-19 Vaccine Acceptance? Evidence From a Nationwide Survey in Japan

**DOI:** 10.1007/s10903-022-01419-4

**Published:** 2022-11-10

**Authors:** Yuanyuan Teng, Tomoya Hanibuchi, Tomoki Nakaya

**Affiliations:** 1 Center for Northeast Asian Studies, Tohoku University, 41 Kawauchi, Aoba-ku, 980-8576 Sendai, Miyagi Japan; 2 Graduate School of Environmental Studies, Tohoku University, Aoba-468-1 Aramaki, Aoba-ku, 980-0845 Sendai, Miyagi Japan

**Correction to: Journal of Immigrant and Minority Health**

10.1007/s10903-022-01402-z

The original version of this article unfortunately missed to include Acknowledgement section.

Therefore, the complete section is given here:

**Acknowledgements**: This work was supported by Promoting Grants for Research Toward Resilient Society 2021 (Tohoku University) and Ensemble Grants for Early Career Researchers 2021 (Tohoku University).

The original article has been corrected.

